# GSTP1 Ile105Val polymorphism correlates with progression-free survival in MCRC patients treated with or without irinotecan: a study of the Dutch Colorectal Cancer Group

**DOI:** 10.1038/sj.bjc.6604654

**Published:** 2008-09-16

**Authors:** D M Kweekel, M Koopman, N F Antonini, T Van der Straaten, J W R Nortier, H Gelderblom, C J A Punt, H-J Guchelaar

**Affiliations:** 1 Department of Clinical Pharmacy and Toxicology, Leiden University Medical Center, Leiden, The Netherlands; 2 Department of Oncology, University Nijmegen Medical Center, Nijmegen, The Netherlands; 3 Biometrics Department, Netherlands Cancer Institute (NKI), Amsterdam, The Netherlands; 4 Department of Clinical Oncology, Leiden University Medical Center, Leiden, The Netherlands

**Keywords:** irinotecan, GSTP1, capecitabine, colorectal cancer, survival, toxicity

## Abstract

A Valine residue at position 105 of the GSTP1 protein results in decreased enzyme activity. As nuclear GSTP1 activity decreases irinotecan cytotoxicity, Val-allele carriers may benefit more from irinotecan chemotherapy. Our aim was to investigate the association of GSTP1 genotype with treatment outcome of irinotecan. Progression-free survival (PFS) and toxicity were determined in 267 metastatic colorectal cancer (MCRC) patients who were treated with first-line capecitabine (CAP) plus irinotecan (CAPIRI), or CAP single agent in a prospective randomised phase III trial (CAIRO). GSTP1 genotype was determined by Pyrosequencing. Patients receiving CAP showed a PFS of 6.6 (Ile/Ile), 6.0 (Ile/Val) and 6.5 months (Val/Val); compared to 7.0 (Ile/Ile), 8.8 (Ile/Val) and 9.2 months (Val/Val) with CAPIRI. Median PFS was 2.7 months longer in Val-allele carriers treated with CAPIRI compared to CAP (*P*=0.005). Patients with the Ile/Ile genotype showed similar PFS with CAPIRI and CAP (7.0 compared to 6.6 months, *P*=0.972). Toxicity did not differ significantly among genotypes. GSTP1 codon 105 polymorphism may be predictive for the response to irinotecan-based chemotherapy in patients with MCRC, with the Val-allele being associated with a better outcome. Ile/Ile genotype patients do not appear to benefit from the addition of irinotecan to CAP.

The topo-isomerase I inhibitor irinotecan is an effective antitumour agent that is frequently used in the treatment of patients with metastatic colorectal cancer (MCRC) (Punt, 2004). Irinotecan may lead to serious and potentially life-threatening side effects, and not all patients respond to irinotecan. Therefore, pharmacokinetic and pharmacogenomic studies are warranted to predict which patients are most likely to benefit from irinotecan-based chemotherapy (Pander *et al*, 2007).

The metabolic fate of irinotecan has been extensively studied. Briefly, the (nearly) inactive prodrug irinotecan is metabolised to SN-38 by the carboxylesterases in the liver. SN-38 is highly active and inhibits topo-isomerase I, thereby interfering with DNA replication and ultimately leading to cell death of dividing cells.

The active metabolite SN-38 is glucuronidated to the inactive SN-38-G by uridine diphosphate glucuronosyl transferases (UGTs) (Iyer *et al*, 1998). An alternative metabolic route leads to the formation of APC (7-ethyl-10-[4-N-(5-aminopentanoic acid)-1-piperidino]-carbonyloxycamptothecin) by CYP3A4 and CYP3A5. Differences in systemic exposure to irinotecan and SN-38 are to some extent explained by genetic variations in UGT1A1 (Paoluzzi *et al*, 2004) but not CYP3A (de Jong *et al*, 2006). Several membrane efflux pumps have a function in the bio-distribution of irinotecan and its metabolites, of which ABCB1 and ABCC2 are of particular interest (de Jong *et al*, 2007; Kweekel *et al*, 2008). Much effort has also been made to explore associations between polymorphisms in UGTs and either toxicity or antitumour response of irinotecan. Although there is evidence that a decreased glucuronidation (UGT1A1^*^28 polymorphism) causes an increased risk of neutropenia and neutropenic fever (Mathijssen *et al*, 2003; Kweekel *et al*, 2008), data regarding the antitumour effects of irinotecan-based chemotherapy are contradicting. This may be because of the fact that systemic irinotecan levels are different from intratumoural concentrations, and also because of differences in the capacity of a tumour to metabolise or excrete the drug. An elevated expression of UGTs or membrane pumps may prevent or limit irinotecan-induced cell death in cancerous tissue, by causing a resistant phenotype. In this respect, the intratumoural expression of a wide range of proteins has been studied.

One of the proteins with an elevated expression in multidrug-resistant cell lines is glutathione-*S*-transferase pi (GSTP1) (Tew, 1994). GSTP1 has multiple functions, of which the enzymatic conjugation of glutathione (GSH) to xenobiotics is the most widely known. Irinotecan or SN-38 are not known as substrates for this conjugation reaction, but *in vitro* experiments show that if nuclear expression of GSTP1 is decreased using a mushroom lectin, colonic HCT8 cells are more sensitive to irinotecan (Goto *et al*, 2002). Therefore, GSTP1 may have an important function in the clinical efficacy of irinotecan and/or its metabolites. In addition, GSTP1 is a polymorphic gene, and the codon 105 polymorphism (313A>G, or Ile105Val) influences the geometry of the hydrophobic-binding site of GSTP1 enzyme (Zimniak *et al*, 1994). This results in differences in enzyme specificity and activity (Zimniak *et al*, 1994; Watson *et al*, 1998), which may influence irinotecan cytotoxicity. To our knowledge, the association between GSTP1 polymorphisms and clinical outcome in patients treated with irinotecan has not yet been explored in detail. Therefore, the aim of this study was to investigate the associations of GSTP1 Ile105Val with progression-free survival (PFS) of MCRC patients treated with irinotecan-containing chemotherapy.

## Patients and methods

### Subjects

Blood samples were obtained from patients enrolled in a multicenter phase III trial, the CAIRO study of the Dutch Colorectal Cancer Group (DCCG), of which the results have been published recently (Koopman *et al*, 2007). We refer to this article for a detailed description of eligibility criteria and response or toxicity evaluation. In summary, patients with MCRC were allocated to sequential (regimen A) or combination treatment (regimen B). Regimen A consisted of first-line capecitabine (1250 mg m^−2^ day^−1^ b.i.d. on days 1–14, every 3 weeks: CAP), second-line irinotecan (350 mg m^−2^ day^−1^ on day 1 every 3 weeks) and third-line CAP (1000 mg m^−2^ day^−1^ b.i.d. on days 1–14 every 3 weeks) plus oxaliplatin (130 mg m^−2^ on day 1 every 3 weeks). Regimen B consisted of first-line CAP (1000 mg m^−2^ day^−1^ b.i.d. on days 1–14, every 3 weeks) plus irinotecan (250 mg m^−2^ day^−1^ on day 1 every 3 weeks: CAPIRI), followed by second-line CAP plus oxaliplatin (as described in regimen A).

Dose reductions were performed for CAP in case of grades 2–4 toxicity as described previously (Van Cutsem *et al*, 2001). An initial irinotecan dose reduction to 80% in cycle 1 was recommended in case of: age >70 years, WHO performance status 2 and/or serum bilirubin 1–1.5 × upper normal limit. If well tolerated, the dose was escalated to 100% in subsequent cycles. In all patients, the irinotecan dose was reduced with 25% relative to the previous cycle in case of any grades 3–4 toxicity with the exception of nausea/vomiting without adequate prophylaxis. If these toxicities recurred despite dose reduction, the dose was reduced to 50% and upon next recurrence the treatment was discontinued. Prophylactic use of haematological growth factors or loperamide was not permitted. The accrual period was from January 2003 to December 2004, and EDTA blood samples for genotyping were collected from December 2003 to March 2005 after a protocol amendment. The objective of this amendment was to perform genetic association studies regarding antitumour response and toxicity. The study protocol and the amendment were approved by the local ethical committees. Written informed consent was obtained from all patients participating in the genetic association study before blood collection. DNA was obtained from 267 patients (regimen B: 141 subjects; regimen A: 126). Tumour evaluation was performed every three cycles according to the RECIST criteria (Therasse *et al*, 2000), toxicity according to US National Cancer Institute Common Toxicity Criteria, version 2.0. All results were blinded with respect to genotype.

### Genotyping

Genomic DNA was isolated from peripheral blood cells (MagnaPure Total Nucleic Acid Isolation Kit I on MagnaPure LC (Roche Diagnostics, Mannheim, Germany)). Chromosomal DNA was quantified using Nanodrop (Isogen, IJsselstein, The Netherlands) and diluted to 10 ng *μ*l^−1^.

Primers for the GSTP1 Ile105Val polymorphism (rs1695) and pyrosequence materials were obtained from Isogen Life Sciences (IJsselstein, The Netherlands), Sepharose beads from Amersham (Uppsala, Sweden). PCR reactions were performed using Hotstart PCR mastermix (Qiagen, Hilden, Germany) on the MyCycler (Biorad, Veenendaal, The Netherlands). Pyrosequence analysis was performed on a Pyrosequencer 96MA (Biotage, Sweden). PCR reactions were as follows: each reaction contained 10 ng of DNA, and 5 pmol of each PCR primer (forward: 5′-AGGACCTCCGCTGCAAATAC-3′, reverse 5′-CTGGTGCAGATGCTCACATAGTT-3′) in a total of 12 *μ*l. Cycle conditions were as follows: initial denaturation for 15 min at 95°C, 35 cycles of 95°C−55°C−72°C each for 30 s, ended by 10 min at 72°C. The pyrosequence reactions were performed according to the manufacturer's protocol. The sequence to analyse was **A/G**TCTCCCTCAT using the forward sequence primer 5′-CTCCGCTGCAAATAC-3′.

### Statistics

Possible associations of GSTP1 genotype with the incidence of diarrhoea grades 3–4, febrile neutropenia and overall grades 3–4 toxicity according to the genotype were tested with a Fisher's exact test. The PFS was calculated from the date of randomisation to the first observation of disease progression or death from any cause reported after first-line treatment. The PFS curves were estimated using the Kaplan–Meier method and compared using the log-rank test. Multivariate analysis of PFS was performed by means of a Cox proportional hazard model. All tests were two-sided and *P*-values <0.05 were considered significant. All follow up data received before March 2008 were included in this analysis, representing a median follow up of 41 months. All analyses were performed using SAS 9.1 and S-plus 6.2 software.

## Results

Patient characteristics are shown in Table 1. Patient ethnicity was not recorded but the vast majority of patients were of Caucasian descent. Genotyping the Ile105Val polymorphism was successful in all 267 patients (100%). Overall genotype frequencies for the GSTP1 Ile105Val polymorphism were as follows: Ile/Ile 107(40.3%), Ile/Val 129(48.1%) and Val/Val 31(11.6%). The genotype distribution was in Hardy–Weinberg equilibrium and the allele frequencies (Ile 0.64, Val 0.36) were found in concordance with HapMap data of Caucasian individuals published online (http://www.ncbi.nlm.nih.gov/SNP/snp_ref.cgi?rs=1695).

### Clinical efficacy

**Figure 1:**
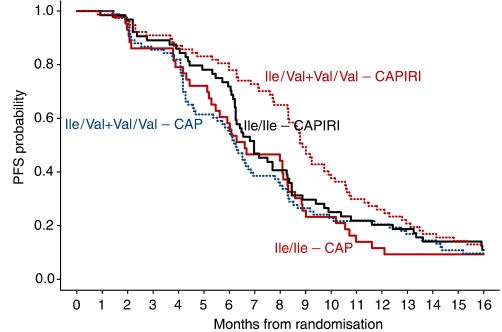
Kaplan–Meier plot of progression-free survival (PFS). Solid lines: Ile/Ile, dotted lines: Ile/Val+Val/Val. If we compare median PFS of Ile/Ile patients receiving CAP with those receiving CAPIRI, we see a nonsignificant difference (*P*=0.972), whereas median PFS of Val carriers is significantly better in the CAPIRI regimen (*P*<0.005). The full-colour version is available online.

All 267 patients were evaluable for analysis of PFS: 126 patients received first-line chemotherapy with CAP (regimen A), and 141 patients received first-line combination therapy with irinotecan and CAP (CAPIRI, regimen B). For each separate genotype, we compared PFS of patients receiving CAP with patients receiving CAPIRI (Table 2, Figure 1). Overall, median PFS was longer for patients receiving CAPIRI: 8.3 (95% confidence intervals (CI): 7.6–8.8) months compared to 6.3 months (95% CI: 5.8–6.9, CAP). This survival benefit from CAPIRI treatment was observed in patients of the Ile/Val and Val/Val genotypes, but not in patients with the Ile/Ile genotype. The median PFS for the separate genotypes receiving CAP were as follows: 6.6 months (Ile/Ile, *n*=43), 6.0 months (Ile/Val, *n*=65) and 6.5 months (Val/Val, *n*=18), *P*=0.886. In the CAPIRI regimen, median PFS were 7.0 months (Ile/Ile, *n*=64), 8.8 months (Ile/Val, *n*=64) and 9.2 months (Val/Val, *n*=13), *P*=0.078. Ile/Ile homozygotes receiving CAP had a similar median PFS compared to Val-allele carriers (median 6.2 months, *P*=0.647). However, the Ile/Ile genotype receiving CAPIRI had a significantly lower median PFS compared to the other genotypes (median 7.0 months compared to 8.9 months, *P*=0.037). Multivariate Cox regression analysis reveals that PFS for the Ile/Ile genotype is not significantly different depending on the use of CAP or CAPIRI (*P*=0.972), which is in contrast to Val-allele carriers (*P*=0.005). This result suggests that Ile/Ile individuals do not benefit from the addition of irinotecan to CAP.

### Toxicity

As was expected, the overall incidence of grades 3–4 diarrhoea and febrile neutropenia was higher in patients treated with CAPIRI (26.2%) compared to CAP (7.1%, *P*<0.001).

There were no statistically significant differences between GSTP1 genotypes in the incidence of overall grades 3–4 toxicity or grades 3–4 diarrhoea with CAP (Table 3).

## Discussion

This is the largest study to date that investigates a possible association of the GSTP1 codon 105 polymorphism with response and toxicity to irinotecan. We demonstrate that, as opposed to the overall study population, Ile/Ile carriers do not benefit from the addition of irinotecan to CAP. A number of studies have been conducted to investigate the GSTP1 polymorphism with respect to the treatment effects of other chemotherapeutic compounds, such as busulfan (Zwaveling *et al*, 2008), melphalan (Kuhne *et al*, 2008), cyclophosphamide (Zhong *et al*, 2006), oxaliplatin (Stoehlmacher *et al*, 2002; Ruzzo *et al*, 2007) and cisplatin (Goekkurt *et al*, 2006). *In vitro* data (Goto *et al*, 2002) have shown that the presence of GSTP1 in the cell nucleus protects the cell from irinotecan-induced apoptosis. As irinotecan or its active metabolites are not known to be substrates of the GSTP1 enzyme, we hypothesise that GSH conjugation of reactive oxygen species originating from irinotecan exposure may contribute to this effect. The enzymatic activity of GSTP1 Val105 was found to be lower compared to GSTP1 Ile105 (Watson *et al*, 1998), and patients with Val/Val and Ile/Val may therefore benefit more from irinotecan-induced oxidative damage, compared to the Ile/Ile genotype. Our findings now for the first time suggest a possible relationship between the GSTP1 genotype and irinotecan efficacy in cancer patients.

Based on the results of this study, one may question the use of irinotecan in MCRC patients with the Ile/Ile genotype, as no benefit in median PFS was observed while the incidence of irinotecan-associated toxicity was unchanged.

A recent (FOCUS) study reports a multimarker analysis of, among others, GSTP1 codon 105, in a large set of patients newly presenting with MCRC (Braun *et al*, 2008). Patient characteristics seem comparable with this study, but instead of CAP, 5-fluorouracil (5-FU) was used. The GSTP1 genotype distribution in the FOCUS study was different from this study (with a remarkable 43% of patients being of the homozygote mutant, GG, genotype). Although the authors do not provide a detailed description of GSTP1 associations with time to first-line treatment failure, it is suggested that this genotype is not associated with differences in response between 5-FU plus irinotecan treatment and 5-FU alone. This finding is in contrast to the current results, but may be related to the use of 5-FU instead of CAP (Seymour *et al*, 2007). In addition, both studies report different toxicity patterns and –incidence relating to irinotecan (Koopman *et al*, 2006). The same unknown mechanisms underlying these differences may also affect the association between GSTP1 genotypes and PFS.

A possible limitation to this study is the fact that samples were obtained at a median of 5 months after inclusion, that is, most patients who donated a sample were either in first- or second-line therapy. The overall genotype distribution is not different from data on Caucasians with colorectal carcinoma (Ile/Ile 47, Ile/Val 46 and Val/Val 6%) (Skjelbred *et al*, 2007). A similar distribution is also found in CAPIRI patients, but not in those receiving CAP. This may be a sign of selection bias; however, the genotype distributions in both regimens are in Hardy–Weinberg equilibrium, and the baseline characteristics of the patients randomised to CAP do not differ significantly from the overall patient characteristics of the complete cohort of the original study (*P*>0.2 for all characteristics, data not shown) (Koopman *et al*, 2007). In addition, a recent study found no association of GSTP1 genotype with overall survival or PFS in MCRC patients (Le Morvan *et al*, 2007), indicating that GSTP1 is not a prognostic marker. Therefore, selection bias seems unlikely.

In summary, the results of this study suggest that patients with the Ile/Ile genotype do not benefit, in terms of PFS, from the addition of irinotecan to CAP, in contrast to the Ile/Val and Val/Val genotype. This is the first study reporting an association of the GSTP1 codon 105 variants with the clinical efficacy of irinotecan. If confirmed, this observation has important implications for the selection of MCRC patients who are eligible for treatment with irinotecan.

## Figures and Tables

**Table 1:** Patient characteristics

**Characteristics**	**CAP Ile/Ile, *N*=43 (34%)**	**Ile/Val, *N*=65 (52%)**	**Val/Val, *N*=18 (14%)**	**All patients, *N*=126**
*Age (years)*				
Median	60	62	64	61.5
Range	(36–78)	(27–78)	(47–78)	(27–78)
>70	8 (19%)	12 (19%)	3 (17%)	3 (17%)
				
*Gender*				
Male (%)	27 (63%)	39 (60%)	10 (56%)	76 (60%)
				
*WHO performance status (at start therapy)*				
0–1	41 (95%)	60 (93%)	18 (100%)	119 (94%)
2	2 (5%)	5 (8%)	0	7 (6%)
				
Prior adjuvant therapy	5 (12%)	6 (9%)	2 (11%)	13 (10%)
				
*Predominant localisation of metastases (at inclusion)*				
Liver	27 (63%)	44 (68%)	12 (67%)	83 (66%)
Extrahepatic	15 (35%)	18 (28%)	6 (33%)	39 (31%)
Unknown	1 (2%)	3 (5%)	—	4 (3%)
				
*Site of primary*				
Colon and sigmoid	31 (72%)	42 (65%)	12 (67%)	85 (68%)
Rectum	11 (26%)	23 (35%)	6 (33%)	40 (32%)
Multiple tumours	1 (2%)	—	—	1 (<1%)
				
*Serum LDH*				
Normal	32 (74%)	39 (60%)	11 (61%)	82 (65%)
⩾ULN	11 (26%)	26 (40%)	7 (39%)	44 (35%)
				
**Characteristics**	**CAPIRI Ile/Ile, *N*=64 (45%)**	**Ile/Val, *N*=64 (45%)**	**Val/Val, *N*=13 (10%)**	**All patients, *N*=141**
*Age (years)*				
Median	61.5	63	60	62
Range	41–81	37–78	45–72	37–81
>70	12 (19%)	13 (20%)	1 (8%)	26 (18%)
				
*Gender*				
Male (%)	38 (59%)	40 (63%)	9 (69%)	87 (62%)
				
*WHO performance status (at start therapy)*				
0–1	59 (92%)	60 (94%)	13 (100%)	132 (94%)
2	5 (8%)	4 (6%)	—	9 (6%)
				
Prior adjuvant therapy	10 (16%)	7 (11%)	1 (8%)	18 (13%)
				
*Predominant localisation of metastases (at inclusion)*				
Liver	42 (66%)	46 (72%)	10 (77%)	98 (70%)
Extrahepatic	22 (34%)	18 (28%)	3 (23%)	43 (30%)
				
*Site of primary*				
Colon and sigmoid	37 (58%)	41 (64%)	7 (54%)	85 (60%)
Rectum	27 (42%)	23 (36%)	6 (46%)	56 (40%)
				
*Serum LDH*				
Normal	44 (69%)	39 (61%)	10 (77%)	93 (66%)
⩾ULN	20 (31%)	25 (39%)	3 (23%)	48 (34%)

CAP=capecitabine monotherapy; CAPIRI=capecitabine plus irinotecan combination therapy.

**Table 2:** Progression-free survival of patients using CAP or CAPIRI, according to GSTP1 genotype

**Genotype**	**CAP PFS in months (95% CI)**	***P*-value**	**CAPIRI PFS in months (95% CI)**	***P*-value**
*GSTP1*				
All genotypes (*n*=267)	6.3 (5.8;6.9)	—	8.3 (7.6;8.8)	—
Ile/Ile (*n*=107)	6.6 (5.5;8.3)	Reference	7.0 (6.2;8.3)	Reference
Ile/Val (*n*=129)	6.0 (4.4;7.8)	0.697	8.8 (8.3;10.0)	0.025
Val/Val (*n*=31)	6.5 (6.1;8.3)	0.661	9.2 (7.7;10.6)	0.696
Ile/Val+Val/Val (*n*=160)	6.2 (5.4;7.9)	0.647	8.9 (8.3;9.9)	0.037
Ile/Ile vs Ile/Val *vs* Val/Val	—	0.886	—	0.078
	**Ile/Ile**	**Ile/Val+Val/Val**	**All genotypes**	
*Risk*				
Hazard ratio for PFS CAPIRI *vs* CAP	0.99 (0.67;1.48)	0.63 (0.46;0.87)	0.77 (0.56;0.93)	
*P*-value	0.972	0.005	0.012	

Median PFS, and *P*-values resulting from Cox regression analysis with serum LDH as a covariate. PFS=progression-free survival; CAP=single-agent capecitabine; CAPIRI=irinotecan combination therapy with capecitabine.

**Table 3:** Grades 3–4 toxicity with CAP and CAPIRI treatment

**GSTP1-capecitabine single agent**	**IleIle, *n*=43**	**IleVal, *n*=65**	**ValVal, *n*=18**	**Total, *n*=126**	***P*-value**
Overall grades 3–4	19 (44.2%)	29 (44.6%)	5 (27.8%)	53 (42.1%)	*P*=0.436^F^
Diarrhoea grades 3–4	4 (9.3%)	4 (6.2%)	1 (5.6%)	9 (7.1%)	*P*=0.889^F^
					
**GSTP1-capecitabine plus irinotecan**	**IleIle, *n*=64**	**IleVal, *n*=64**	**ValVal, *n*=13**	**Total, *n*=141**	***P*-value**
Overall grades 3–4	36 (56.3%)	37 (57.8%)	7 (53.8%)	80 (56.7%)	*P*=0.960^C^
Diarrhoea grades 3–4	14 (21.9%)	18 (28.1%)	2 (15.4%)	34 (24.1%)	*P*=0.592^F^
Febrile neutropenia grades 3–4	5 (7.8%)	3 (4.7%)	0 (0.0%)	8 (5.7%)	*P*=0.655^F^
Diarrhoea grades 3–4 or febrile neutropenia	16 (25.0%)	19 (29.7%)	2 (15.4%)	37 (26.2%)	*P*=0.634^F^

Values in this table describe worst grade toxicity, occurring during first-line capecitabine (single agent) or irinotecan plus capecitabine (combination) treatment. F=Fisher's exact test; C=*χ*^2^ test. In capecitabine single-agent users, no febrile neutropenia was recorded.
